# Environmental Justice and the NIEHS

**DOI:** 10.1289/ehp.114-a686

**Published:** 2006-12

**Authors:** David A. Schwartz

**Affiliations:** Director, NIEHS and NTP, E-mail: david.schwartz@niehs.nih.gov

The NIEHS relationship with the environmental justice community is much like that of a family—we have a long history, we are integrally connected, and unfortunately, we don’t always communicate well. I recently met with our environmental justice grantees, and during our conversations, I realized that my own failure to communicate in the accepted terminology of the environmental justice community may have led to a misunderstanding of my commitment to these issues. The failure was unintentional; embracing diversity is such an inherent part of my own life that I may have taken for granted that my commitment to this principle would be obvious. Diversity in my own family has created depth, strength, and opportunity, and I feel the same is true of the environmental justice community’s interconnectedness with the NIEHS. Nevertheless, I now know it is incumbent on me to clearly describe my views regarding environmental justice and explicitly state how we, as an institute, plan to move forward in this relationship.

Diversity in my own family has created depth, strength, and opportunity, and I feel the same is true of the environmental justice community’s interconnectedness with the NIEHS.

Although I admit that I’ve sometimes found the terms “environmental justice” and “community-based participatory research,” as they’ve been defined by the constituency, to be unnecessarily constraining, I strongly believe in the concepts and in their need to be integrated into what we do. In fact, I believe that these concepts form the core of our mission at this particular institute. It is our obligation to support research that produces findings that will inform environmental justice for all people. And we’ve done this well. I applaud the exceptional work of the NIEHS environmental justice grantees; there could be no group of more talented, dedicated, and passionate individuals. But I think the time has come to take our approach to environmental justice issues to a new level.

Just as in families, in the scientific and outreach communities we can sometimes become too comfortable or even complacent, resting on the expected, the traditional, the feeling that “this is how it should be done” because “this is how it has always been done.” Expectations become implied, though perhaps not effectively understood. And this can have the effect of impeding real growth. Environmental justice is too important to suffer that fate. We must take this opportunity to reexamine our efforts, refocus our direction, and reinforce our commitment to the best possible outcomes in this area.

I strongly believe that environmental justice and community-based participatory research should not be carved out to stand alone, but rather that they should be among the most integral, acknowledged, and accepted components of our population-based research. This requires that they be incorporated by both language and action into the tools of our trade. As a first step toward this end, we have explicitly confirmed the importance of environmental justice research and our commitment to it in the NIEHS Strategic Plan. Goal IV of the plan states: “The NIEHS has taken a lead role both in investigating the environmental influences on [disease] conditions in minority and socioeconomically disadvantaged populations and in developing tools and strategies that will prove effective for reducing health disparities. We will continue to support research, both domestically and globally, that can offer important insights into how to reduce exposures and disease incidence in these community settings.” In fact, this goal serves as one of the primary motivating factors for the population-based research we have initiated in New Orleans to study and prevent childhood asthma.

And we will go further. Our environmental justice grantees have helped me to realize anew that although our actions have power, so too do our words. Until such time as environmental justice is achieved, there will continue to be a need to expressly state its importance. Our institute must be a standard bearer in this area. And as its director, I personally will continue to reiterate the needs, goals, and importance of environmental justice, as well as my deep support for it, to the broader scientific community and the world at large. My hope is that our institute and the environmental justice community will continue to come together to acknowledge when we have misunderstood each other, have frank and honest discussions about what it is that we need from each other, and move forward in strong support of each other. After all, that’s what families do.

## Figures and Tables

**Figure:**
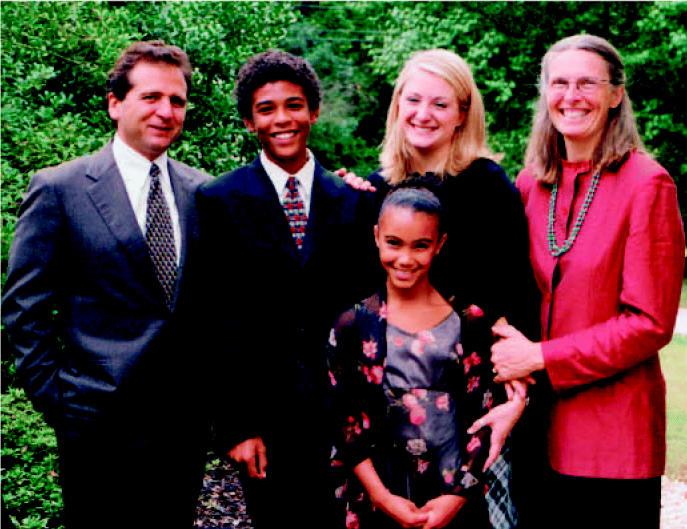
(left to right) David, Samuel, Kiera, and Tziporah Schwartz, Louise Sparks

